# Supplementary material for: High Expression of AhR and Environmental Pollution as AhR-Linked Ligands Impact on Oncogenic Signaling Pathways in Western Patients with Gastric Cancer—A Pilot Study
Source: Biomedicines. 2024 Aug 20;12(8):1905. doi: 10.3390/biomedicines12081905 (PMC11351739; doi:10.3390/biomedicines12081905)
Supplement: Supplementary file 1 [file biomedicines-12-01905-s001.zip › biomedicines-3085459-supplementary.pdf]

**Sup Table S1. Relationship between *AhR* mRNA expression and clinical biological parameters in a series of 29 gastric cancers including diffuse- and intestinal subtypes.**
Median range of gene mRNA expression levels; p value (Mann Whitney). ND, not determined

| **All Tumors n=29** | | **Diffuse sub-type n=13** | | **Intestinal sub-type n=16** | |
|---|---|---|---|---|---|
| | ***AhR*** | | ***AhR*** | | ***AhR*** |
| **Gender**<br>**Male (n=13)**<br>**Female (n=16)** | p=0.51<br>1.54 (0.55-3.33)<br>1.35 (0.65-3.53) | **Gender**<br>**Male (n=6)**<br>**Female (n=7)** | p=0.81<br>1.51 (0.87-3.08)<br>1.58 (1.22-2.01) | **Gender**<br>**Male (n=7)**<br>**Female (n=9)** | p=0.19<br>1.71 (0.89-2.84)<br>1.16 (0.89-1.83) |
| **Age**<br>**<60 years (n=9)**<br>**>60 years (n=20)** | p=0.82<br>1.94 (0.55-3.35)<br>1.80 (0.65-3.53) | **Age**<br>**<60 years (n=8)**<br>**>60 years (n=5)** | p=0.72<br>1.51 (0.87-2.01)<br>1.58 (1.22-3.08) | **Age**<br>**<60 years (n=1)**<br>**>60 years (n=15)** | p=0.80<br>1.38 (0.89-1.83)<br>1.16 (0.90-2.84) |
| **Smoking**<br>**negative (n=12)**<br>**positive (n=10)** | p=0.85<br>1.77 (0.55-3.35)<br>1.85 (0.82-3.53) | **Smoking**<br>**negative (n=4)**<br>**positive (n=3)** | p=0.63<br>1.51 (0.87-1.63)<br>1.58 (1.27-2.01) | **Smoking**<br>**negative (n=8)**<br>**positive (n=7)** | p=0.74<br>1.19 (0.89-1.71)<br>0.96 (0.9-1.55) |
| **Tumor invasion T**<br>**T1-T2 (n=6)**<br>**T3-T4 (n=23)** | p=0.21<br>1.45 (0.65-2.86)<br>1.94 (0.55-3.53) | **Tumor invasion**<br>**T1-T2 (n=2)**<br>**T3-T4 (n=11)** | ND<br>1.42 (1.27-1.58)<br>1.53 (0.9-3.1) | **Tumor invasion**<br>**T1-T2 (n=4)**<br>**T3-T4 (n=12)** | ND<br>0.93 (0.9-0.96)<br>1.55 (0.89-2.84) |
| **Lymphatic invasion**<br>**negative (n=11)**<br>**positive (n=17)** | p=0.11<br>1.25 (0.65-3.18)<br>2.11 (0.55-3.53) | **Lymphatic invasion**<br>**negative (n=1)**<br>**positive (n=12)** | ND<br>1.53<br>1.53 (0.87-3.08) | **Lymphatic invasion**<br>**negative (n=10)**<br>**positive (n=5)** | p=0.61<br>1.71 (0.65-3.18)<br>1.94 (1.21-3.53) |
| **Metastasis M**<br>**negative (n=24)**<br>**positive (n=5)** | p=0.92<br>1.96 (0.55-3.53)<br>2.1 (0.82-2.55) | **Metastasis**<br>**negative (n=9)**<br>**positive (n=4)** | p=0.93<br>1.49 (0.87-3.08)<br>1.58 (1.22-2.01) | **Metastasis**<br>**negative (n=15)**<br>**positive (n=1)** | p=0.79<br>1.71 (0.89-2.84)<br>1.19 (1.16-1.55) |
| **TNM**<br>**I-II (n=16)**<br>**III-IV (n=13)** | p=0.30<br>1.77 (0.54-3.35)<br>2.11 (0.82-3.53) | **TNM**<br>**I-II (n=5)**<br>**III-IV (n=8)** | p=0.21<br>1.32 (0.87-1.9)<br>1.6 (1.22-3.08) | **TNM**<br>**I-II (n=11)**<br>**III-IV (n=5)** | p=0.80<br>1.71 (0.89-1.83)<br>1.19 (0.9-2.84) |
| **Vascular invasion**<br>**negative (n=9)**<br>**positive (n=20)** | p=0.14<br>1.25 (0.82-2.96)<br>2.05 (0.55-3.53) | **Vascular invasion**<br>**negative (n=3)**<br>**positive (n=10)** | p>0.9999<br>1.53 (1.32-1.63)<br>1.53 (0.87-3.08) | **Vascular invasion**<br>**negative (n=6)**<br>**positive (n=10)** | p=0.79<br>1.21 (1.18-1.72)<br>1.35 (0.89-2.84) |
| **Neural invasion**<br>**negative (n=6)**<br>**positive (n=23)** | p=0.47<br>1.58 (0.90-2.86)<br>1.94 (0.55-3.53) | **EPN**<br>**negative (n=2)**<br>**positive (n=11)** | ND<br>1.42 (1.27-1.58)<br>1.53 (0.9-3.1) | **EPN**<br>**negative (n= 4)**<br>**positive (n= 12)** | ND<br>0.93 (0.9-0.96)<br>1.55 (0.89-2.84) |
